# Hemoparasites in Wild Birds: A Systematic Review of Their Ecology and Clinical Implications

**DOI:** 10.3390/ani15172570

**Published:** 2025-09-01

**Authors:** Alberto Alvarado-Piqueras, María Teresa Gómez-Muñoz, Bárbara Martín-Maldonado

**Affiliations:** 1 Group of Rehabilitation of the Wild Fauna and Its Habitat, Wildlife Hospital of GREFA, 28220 Majadahonda, Spain; 2 Department of Animal Health, Faculty of Veterinary Sciences, University Complutense of Madrid, Avenida Puerta de Hierro s/n, 28040 Madrid, Spain; mariateresa.gomez.munoz@pdi.ucm.es; 3 Department of Veterinary Medicine, School of Biomedical and Health Sciences, Universidad Europea de Madrid, 28670 Villaviciosa de Odón, Spain; barbara.martin-maldonado@universidadeuropea.es

**Keywords:** hemosporidian, *Plasmodium*, *Haemoproteus*, *Leucocytozoon*, *Trypanosoma*, filarial worms, Apicomplexa, wildlife, pathogenicity

## Abstract

Hemoparasites are blood-borne parasites transmitted by vectors and are commonly found in wild birds worldwide. The most frequently reported genera include *Plasmodium*, *Haemoproteus*, *Leucocytozoon*, and *Trypanosoma*, while filarial worms and other hemoparasites are less frequently reported. Despite their importance, knowledge of their effects on the health of wild birds remains limited. This systematic review summarizes all scientific findings on hemoparasites in wild birds, emphasizing their implications for host health. The literature reveals a high number of articles in this area in recent decades, reflecting a growing concern about hemoparasites in wildlife and their ecological impact. However, studies remain unevenly distributed across regions and bird taxa. Diagnostic limitations, particularly in fieldwork and wildlife rehabilitation, continue to present challenges for the accurate detection and monitoring of these parasites. Nevertheless, molecular tools have advanced our understanding of parasite diversity and host–parasite dynamics. Our work highlights key knowledge gaps and stresses the need for further research to improve the understanding of the role of hemoparasites in avian ecology, particularly concerning environmental change and emerging infectious diseases.

## Simple Summary

Blood parasites transmitted by insects can affect wild birds across the globe, but their impact on bird health is not well understood. This study reviewed existing scientific research to better understand these parasites and how they affect birds in nature. Although more research has been performed in recent years, it is mostly limited to certain regions and types of birds. There are still many challenges in identifying these parasites, especially when studying birds in the wild or at rescue centers. However, modern laboratory tools have helped scientists to learn more about the variety of parasites and how they interact with their bird hosts. This review points out the gaps in current knowledge and calls for more research to understand how these parasites affect bird populations, especially as environmental changes and new diseases continue to emerge. A deeper understanding of these parasites will help to protect bird health and biodiversity in a changing world.

## 1. Introduction

Parasitism can be defined as an interspecific association between two organisms, the parasite and the host. The parasite is metabolically reliant on the host and engages in a mutual exchange of substances [1,2,3]. Hemoparasites are obligatory parasitic organisms that infect the blood cells of vertebrate animals. Vectors transmit them and are currently distributed across all continents [4]. The impact on human and animal health is substantial, with numerous species of hemoparasites recognized for causing diseases ranging from debilitating to fatal. Vector-borne diseases are subject to constant changes due to global warming, species migration, globalization, and vector transportation, among others [5]. Their significance in veterinary medicine is well recognized. However, compared to domestic species, knowledge regarding their biology, transmission, and particularly pathogenesis in wildlife remains limited [6,7].

The most common hemoparasites among wild birds are unicellular protists, such as *Haemoproteus* spp., *Leucocytozoon* spp., *Plasmodium* spp., and *Trypanosoma* spp. [8,9,10,11]. The genera *Haemoproteus*, *Leucocytozoon*, and *Plasmodium* belong to the order Haemospororida within the phylum Apicomplexa. By contrast, the genus *Trypanosoma* is classified under the order Trypanosomatida within the phylum Euglenozoa [12].

One of the main challenges in the study of hemoparasites arises from the vast diversity of species, including hosts, hemoparasites, and even vectors. Furthermore, the high dispersal ability of these avian species facilitates contact between diverse hemoparasites and vectors. This increases the likelihood of interactions with naive hosts, making the study more complex, as these host–parasite relationships constantly evolve [13]. Also, the increased vectorial spread observed in some geographic regions over the past few decades may promote the geographic distribution of vector-borne diseases [14,15].

Wild birds are key agents in the non-anthropogenic spread of pathogens due to their high dispersal capacity [13,16,17]. Although certain well-known hemoparasites, such as *Plasmodium* falciparum and *Trypanosoma brucei*, have been well studied, many others, particularly those affecting wildlife, remain poorly understood [18]. Furthermore, the persistent threat of climate change as a major driver in the emergence and re-emergence of infectious diseases has underscored the growing need to understand the ecological and evolutionary factors that drive the spread of hemoparasites [19,20].

Notably, most countries lack formal wildlife surveillance systems, and the detection of hemoparasites often relies on wildlife rehabilitation centers, which report cases within the constraints of their diagnostic and operational capacities. This fragmented and limited data collection hinders a comprehensive understanding of hemoparasite–host interactions and highlights the urgent need for more systematic and targeted research in this area.

In this context, this systematic review aimed to summarize the scientific information about hemosporidian hemoparasites (*Plasmodium*, *Haemoproteus*, and *Leucocytozoon*) in wild birds and their consequences for host health and to identify gaps in the field of study. Other blood parasites, such as filarial worms or *Trypanosoma* spp., were considered only when reported in the same studies addressing hemosporidians but were not the primary focus of the search strategy.

## 2. Materials and Methods

**Figure 1:**
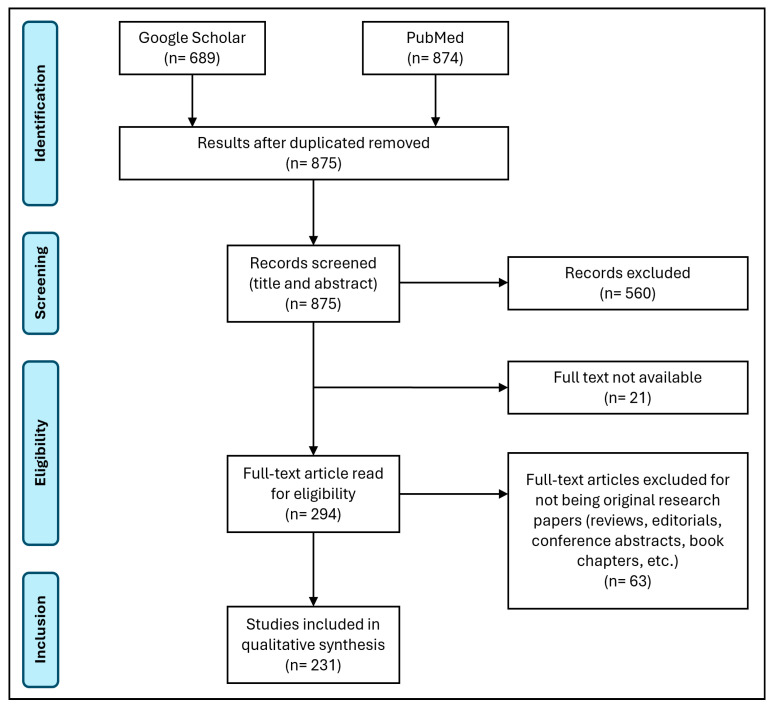
Flow diagram of articles reviewed for the study, following PRISMA 2020 statement [21].

To achieve this, a systematic review was conducted using the PubMed and Google Scholar platforms, focusing on two items: “Hemoparasites” (item 1) and “Wild birds” (item 2). Different search terms for each item were combined by the Boolean operators “OR” and “AND.” For item 1, the search terms were “Hemoparasite”, “Hemosporidia”, “Apicomplexa”, “*Plasmodium*”, “*Trypanosoma*”, “*Haemoproteus*”, and “*Leucocytozoon*.” For item 2, only “wild bird” was employed. The search strategy was designed to target hemosporidian hemoparasites in wild birds; therefore, search terms referring exclusively to non-hemosporidian parasites (e.g., microfilariae) were not included. The search was performed in May 2024, and 876 publications were obtained from the first search. Then, following the PRISMA 2020 statement [21], titles and abstracts were reviewed, and only manuscripts that agreed on both items were included. Studies focused only on hemoparasite vectors, without original data from wild birds, were excluded. Only those that included both vector studies and analyses of hemoparasites in wild birds within the same article were included in this analysis. Other exclusion criteria were full text not available online, non-original studies (reviews, book chapters, and similar), other than peer-reviewed studies, published in non-indexed journals, and language other than English, French, or Spanish (Figure 1). Finally, 231 studies were included in the present review.

## 3. Bibliometric Analysis

**Figure 2:**
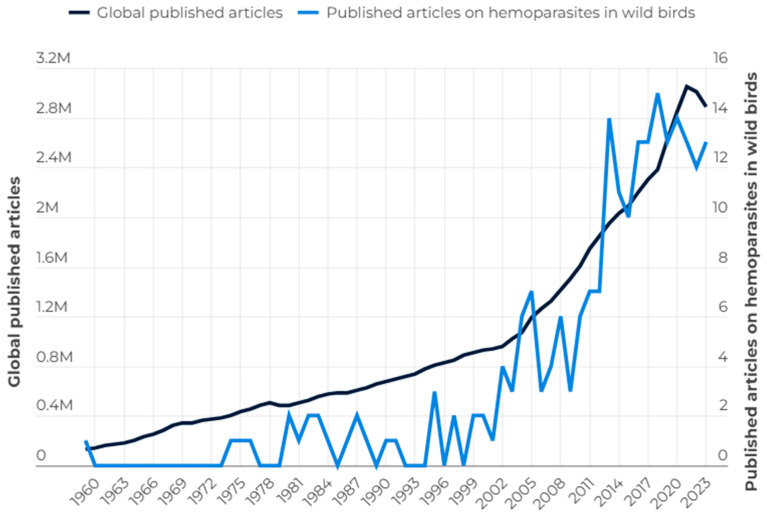
Comparative line chart of annual publications on hemoparasites in wild birds and worldwide scientific publications.

The first study about hemoparasites in wild birds was published in 1959. Since then, the number of publications per year was low until the new millennium. Interest in wildlife research has been increasing, along with the overall number of scientific studies, especially in the last decade, with a mean of 16.8 published original studies on hemoparasites per year (Figure 2). The increase in number of publications after the early 2000s coincides with advances in molecular diagnostics, which have facilitated more sensitive and large-scale parasite detection.

**Figure 3:**
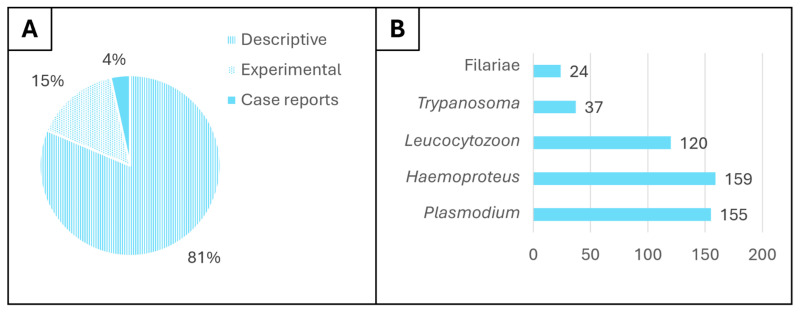
(**A**) Distribution of types of original articles on hemoparasites in wild birds. (**B**) Number of descriptive articles providing data on the main genera of avian hemoparasites. Most of the studies address more than one hemoparasite genus.

Considering only original studies, 188 were descriptive, 35 were experimental, and 8 were case reports (Figure 3A). From the descriptive and case reports publications, 162 were focused on multiple hemoparasite genera (82.7%), while 34 were focused only on one genus (17.3%) (Figure 3B). Overall, 82.4% of the descriptive studies provided data about *Plasmodium*, 84.6% about *Haemoproteus*, 63.8% about *Leucocytozoon*, 19.6% about *Trypanosoma*, and 12.7% about filariae. Other protozoan hemoparasite genera (7.8%) described in wild birds were *Lankesterella*, *Hepatozoon*, *Babesia*, *Atoxoplasma*, *Crithidia*, *Blastocrithidia*, *Herpetomonas*, *Leptomonas*, and *Wallaceina*, and hemoparasite bacteria such as *Borrelia*, *Ehrlichia*, and *Aegyptianella*. It is important to note that many of the studies, particularly older ones, did not report parasite identifications beyond the genus level or did not provide quantitative data for each host species. This limitation prevents the construction of a comprehensive cross-distribution table linking each bird taxon to specific hemoparasite taxa without introducing bias.

**Figure 4:**
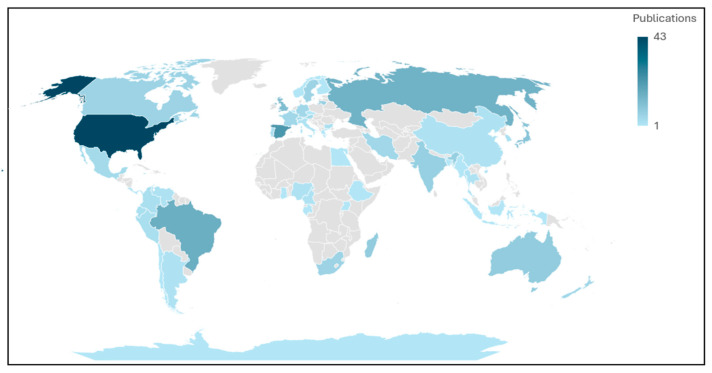
Worldwide distribution of original studies published from 1959 to 2024 about hemoparasites in wild birds.

According to study location, most of the studies were carried out in Europe (37.7%) [22,23,24,25,26,27,28,29,30,31,32,33,34,35,36,37,38,39,40,41,42,43,44,45,46,47,48,49,50,51,52,53,54,55,56,57,58,59,60,61,62,63,64,65,66,67,68,69,70,71,72,73,74,75,76,77,78,79,80,81,82,83,84,85,86,87,88,89,90,91,92,93,94,95,96,97,98,99,100,101,102,103,104,105,106], followed by North America (21.2%) [71,107,108,109,110,111,112,113,114,115,116,117,118,119,120,121,122,123,124,125,126,127,128,129,130,131,132,133,134,135,136,137,138,139,140,141,142,143,144,145,146,147,148,149,150,151,152,153,154,155,156,157,158,159,160,161,162,163], Asia (12.8%) [65,71,143,164,165,166,167,168,169,170,171,172,173,174,175,176,177,178,179,180,181,182,183,184,185,186,187,188,189,190,191], South America (12%) [50,71,153,192,193,194,195,196,197,198,199,200,201,202,203,204,205,206,207,208,209,210,211,212,213,214,215,216,217], Africa (9.7%) [71,218,219,220,221,222,223,224,225,226,227,228,229,230,231,232,233,234,235,236,237], Oceania (4.6%) [71,236,237,238,239,240,241,242,243,244,245,246], Central America (1.7%) [139,247,248], and Antarctica (0.3%) [249]. The United States (18.6%), Spain (9.5%), Brazil (6.9%), Russia (6.5%), the United Kingdom (5.1%), and Japan (4.3%) were the countries with the highest numbers of hemoparasite studies in wild birds in decreasing order (Figure 4). Countries leading in publication output, such as the USA, Spain, and Brazil, often benefit from established ornithological research networks, long-term ecological monitoring programs, and targeted funding for emerging infectious disease research. Conversely, regions with rich avian biodiversity but low research output, such as parts of Africa and Southeast Asia, likely face limitations in research infrastructure, funding allocation, and international collaboration opportunities.

Only 14 of the 231 individual studies included in this systematic review were conducted in more than one country, highlighting the scarcity of multi-country research efforts [26,65,71,83,88,92,101,139,143,153,166,199,232,235]. The predominance of single-country studies also indicates that cross-border collaborations remain underdeveloped, despite the transboundary nature of avian migrations and parasite transmission. Strengthening these collaborations could enhance data comparability, fill geographic gaps, and better integrate parasite ecology into conservation and public health strategies.

**Figure 5:**
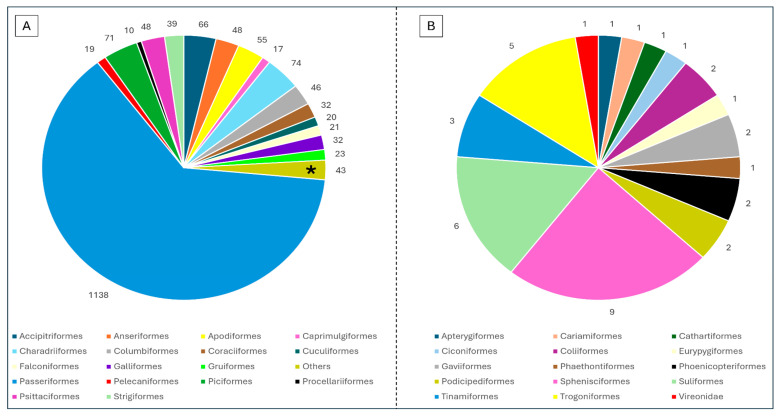
(**A**) Representation of the avian taxonomic orders most assessed over the 188 descriptive studies included in the systematic review. The number of species analyzed within each order is detailed in the graphic’s margin. The asterisk (*) represents the compilation of the least representative orders (with less than 10 species each). (**B**) Details of the proportions of the least representative orders.

More than 1800 avian species have been investigated, representing 176 different families and 33 orders. Among them, the species most assessed was the house sparrow (*Passer domesticus*) (26/188 descriptive studies included this species, 13.8%). The taxonomic focus on certain avian orders, especially Passeriformes (1138/1802 species, 63.2%), likely reflects both sampling convenience and the existence of well-funded monitoring schemes in temperate regions. The proportion of avian orders in which hemoparasites have been assessed is represented in Figure 5, and more details can be found in Supplementary Table S1.

Among the published original studies, 194 were identified as “captured in the field”, 28 were classified as “admitted to recovery centers”, and 9 included both categories.

## 4. Clinical Signs Related to Hemoparasites in Wild Birds

Articles investigating the relationship between hemoparasites and the occurrence of clinical signs were compiled. Studies in which the clinical signs were not supported by statistical analysis were excluded from this analysis. Only 39 of the 231 articles collected were included and classified to assess the impact of hemoparasites within each of the following categories: (1) effects on body condition, mass, and growth; (2) relationship with reproductive success and survival; (3) blood parameters and immune response alterations; (4) effects on activity, body temperature, and behavior; (5) effects on feather quality or growth; and (6) interactions with ectoparasites. Since the first three categories contained the most significant number of compiled articles, they were summarized in tables (see Supplementary Table S2).

### 4.1. Effects on Body Condition, Mass, and Growth

Twenty-six articles performed statistical analyses on body condition index (BCI), mass or growth related to hemoparasites. Among them, most of the articles (14/26) found no statistically significant relationship between hemoparasites and the various variables examined in each study.

Hemoparasites and BCI. The body condition index is a numerical scale used to assess the amount of energy reserves stored and muscle mass development in an individual. This metric is particularly relevant when the specimen size deviates from the species’ expected norm due to underdevelopment, age, or sex, regardless of extrinsic variations such as temperature, latitude [43], or location [144]. Some studies investigating the association between BCI and parasitism intensity (*Plasmodium* spp.) found no evidence of a significant association [56,72,152,244], nor between BCI and the presence/absence of hemoparasites (mainly *Plasmodium* spp.) [159].

However, a recent study reported a significant association between low BCI (2/5) and the presence of *Leucocytozoon*. In barn owls (*Tyto alba*), individuals with a BCI of 2/5 exhibited parasitism rates five times higher than those with a standard BCI of 3/5 [99]. These results suggest that BCI may influence susceptibility to hemoparasites; however, such findings were inconsistent across studies. For instance, Baillie et al. [240] reported a positive relationship between BCI and the presence of *Plasmodium* spp. at one location (Tawharanui, New Zealand) but a negative relationship at another (Hauturu, New Zealand).

Additionally, some studies reported a positive relationship between hemoparasitism and BCI in juvenile individuals, but not in adults, suggesting that age may modulate the effects of parasitic infection on BCI. This pattern was observed by Bichet et al. [79] and Jiménez-Peñuela et al. [77], who suggested that age may play a significant role in the dynamics of parasitism, with higher prevalence observed in adults, possibly due to its chronic nature. Similarly, Gupta et al. [181] found a positive relationship between the presence of *Haemoproteus* spp. and BCI. However, these findings contradicted those of Meixell et al. [147], who reported a negative association between BCI and *Haemoproteus* spp. and *Leucocytozoon* spp., but not for *Plasmodium* spp. infection. Furthermore, they reported a variation in this association with *Leucocytozoon* spp. among host species: negative in northern pintail (*Anas acuta*) and positive in American wigeon (*Mareca americana*). This underscores the importance of monitoring multiple species within the same geographical area to fully understand host–parasite interactions since individual host traits may determine the outcome of the infection [147].

Other studies found no association between hemoparasite presence and BCI [68,214]. These findings suggest that the relationship between BCI and hemoparasitism is likely influenced by a combination of factors, including bird species, age, local environmental conditions, and probably parasite species, highlighting the complexity of these interactions across different ecological contexts. It is important to note that, in many cases, other diseases remained unknown, as co-infections were not specifically investigated. Accounting for all relevant variables is particularly challenging in wild animals, which may contribute to inconsistencies among studies. Also, there was a clear lack of studies examining not only the presence of hemoparasites but also parasitic burden concerning BCI. The few existing ones [226,250] were excluded from this study due to inconsistent data collection methodology.

Hemoparasites and body mass. Body mass measurement eliminates BCI’s subjectivity, providing greater accuracy when combined with other morphometric measurements, such as tarsus length or wing length [251]. Several studies found no statistically significant relationship between the presence or absence of hemoparasites and body mass in experimental studies inoculating hemoparasites such as *Plasmodium elongatum* [75] or *Plasmodium relictum* [62], nor in descriptive studies with *Haemoproteus* spp., *Leucocytozoon* spp., or *Plasmodium* spp. [85], or only *Plasmodium* spp. [159]. Similarly, no significant relationship was observed between parasite intensity and body mass with *P. relictum* [90]. By contrast, Marzal et al. [45] observed lower body mass in infected individuals (*Haemoproteus* spp. and *Plasmodium* spp.) than in uninfected ones. Once again, this discrepancy in results suggests that the relationship between hemoparasitism and body mass may depend on various factors, such as bird or parasite species, type of infection, or environmental conditions, pointing to the need for continued investigation of these interactions to draw more robust conclusions.

Notably, Karell et al. [37] found no association between body mass and *Leucocytozoon* spp. infection in the grey morph, but reduced body mass was observed in the brown morph of the tawny owl (*Strix aluco*) associated with this parasite genus. This underscores the importance of considering morphotype and genetic variation in future studies, a factor that remained relatively unexplored in the studies analyzed [37].

Some studies found a statistically significant relationship between lower body mass in animals with hemoparasite co-infections (*Haemoproteus* spp. and *Plasmodium* spp.) compared to animals without hemoparasites or with only one type of hemoparasite in the blood [31]. These findings suggest that the impact of co-infection on body mass may be variable, potentially influenced by factors such as the species of hemoparasite involved, host species, or ecological characteristics, highlighting the need for further research to understand these interactions better.

Taken together, these findings indicate that hemoparasitic effects on body condition are not entirely idiosyncratic but may follow certain patterns. Co-infection, high parasite loads, and infections occurring in juvenile or nutritionally stressed individuals appear more likely to correlate with reduced BCI or body mass. Morphological variations within species, such as color morphs in owls, may also modulate these impacts, suggesting a role for genetic factors in determining host resilience. Such patterns, while not universal, can guide targeted studies focusing on susceptible host groups or environmental contexts where impacts are most likely to be detected.

### 4.2. Effects on Reproductive Success and Survival

In general, parasitic infections are expected to reduce reproductive performance due to the energetic cost of mounting immune responses or the direct damage caused by the parasite to host tissues and physiological functions [252,253]. However, the studies included in this review revealed a more complex and variable picture. Twelve studies conducted statistical analyses on reproductive success, sex, and survival, based on the premise that survival and reproductive success may be intrinsically connected.

Reproductive parameters. The variability in the parameters examined in the literature was considerable, leading to few definitive conclusions. For instance, Marzal et al. [31] observed that, in western house martins (*Delichon urbicum*), individuals with parasitic co-infections (*Haemoproteus* spp. and *Plasmodium* spp.) produced a higher number of eggs per clutch and successfully reared more fledglings compared to individuals without infections or with a single infection. However, in a subsequent study by the same research group on the same species and geographical region (Spain), greater reproductive success was found in uninfected birds, which initiated laying earlier, had larger clutch sizes, and successfully reared more fledglings than infected birds [45]. This highlights the importance of analyzing co-infections, as they can influence disease severity in complex ways, sometimes reducing pathogenicity through competitive interactions, but often exacerbating disease outcomes. Moreover, age-related data were lacking in these two studies. Reproductive success, parasite prevalence, and co-infection probability tend to increase with age. Age may act as a confounding variable and should be considered in interpreting such associations.

Some authors reported a decrease in fledgling rates related to infection with hemoparasites [72,152]. By contrast, in other studies, no statistically significant differences were reported in the number of eggs laid or the number of nestlings hatched between infected and non-infected female birds. Moreover, infected females showed increased reproductive parameters, such as the number of eggs per clutch, fledglings, and hatching rate [135,146]. In males, no significant alterations were found in spermatozoa following *Plasmodium* spp. inoculation, nor in other reproductive parameters [160].

Survival. Several studies found a negative association between hemoparasitic burden and host survival. For instance, Dadam et al. [73] reported reduced survival in both adult and juvenile house sparrows infected with *P. relictum*, similarly to Townsend et al. [152], who observed decreased survival in *Plasmodium*-infected American crows (*Corvus brachyrhynchos*). However, this effect was not seen with other hemosporidia infections. Hemoparasitism was also linked to increased telomere shortening in common reed warblers (*Acrocephalus arundinaceus*), suggesting a cumulative effect on cellular aging [52]. Furthermore, co-infections appear to intensify the impact on survival, as shown by Pigeault et al. [72], who found that great tits (*Parus major*) co-infected with multiple hemoparasites (*Plasmodium* spp., *Haemoproteus* spp., and *Leucocytozoon* spp.) had lower survival rates than those with single infections. By contrast, an experimental study by Ilgūnas et al. [76] found no association between *Plasmodium elongatum* experimental infection and mortality in common starlings (*Sturnus vulgaris*) and common crossbills (*Loxia curvirostra*). These contrasting findings highlight the variability in the effects of hemoparasitism on survival, which may depend on factors such as host species, hemoparasite species, and co-infection [29].

Despite the variability among species and study designs, two recurrent patterns stand out. First, co-infections are more often associated with altered reproductive parameters, possibly due to the combined energetic demands of multiple immune responses. Second, early breeding and higher fledging success tend to occur in uninfected individuals in several passerine species, suggesting that infection may delay or reduce reproductive output in some contexts. Linking reproductive metrics with detailed parasitological data, including infection stage and intensity, will be critical to determine causality and inform conservation measures for vulnerable populations.

### 4.3. Blood Parameters and Immune Response Alterations

Alterations in hematocrit, packed cell volume (PCV), and hemoglobin. Establishing an increase or decrease in hematocrit levels or PCV may be complex as a pathological event. One of the most likely scenarios associates an increase in these hematological parameters with dehydration [254], while a decrease could be linked to blood loss, like hemorrhage or hemolysis [255].

Although some studies found no relationship between hemoparasitism and hematocrit [90], PCV [79], and hemoglobin [104], other authors observed a statistically significant drop in hematocrit level or PCV following experimental infection by different species of *Plasmodium* [76,111]. For example, wild turkeys (*Meleagris gallopavo*) showed a 20% decrease in mean PCV on day 25 post-infection with *Plasmodium hermani* [111]. This decrease was consistent with the pathophysiology of *Plasmodium*, in which the parasite develops within and ultimately destroys red blood cells during its life cycle, leading to anemia and consequently reduced hematocrit values [99,152,176]. By contrast, other studies found that *Plasmodium* spp. infection was associated with increased hematocrit values, whereas infections with *Haemoproteus* spp. or *Leucocytozoon* spp. were unrelated to such alterations [176].

The study by Jenkins et al. [56] on great tits (n = 67) highlighted the importance of quantifying hemoparasitism and considering the different locations used in the study. While no general relation was found between hematocrit values and parasitic burden, individuals infected with *P. relictum* from Dorigny (Switzerland) exhibited lower hematocrit levels compared to those who were free of the hemoparasite.

As in previous analyses, the response to hemoparasitism varied across different species. For instance, in the study by Sijbranda et al. [244], the PCV of North Island robins (*Petroica longipes*) infected with *Plasmodium* spp. was significantly lower than that of uninfected individuals. However, no relationship was observed between parasitic burden and other species studied. Furthermore, it is essential to assess the influence of co-infection in hemoparasite studies, as shown in the work by Marzal et al. [31], where they reported higher hematocrit levels in western house martin with dual infections (*Haemoproteus* spp. and *Plasmodium* spp.). Notably, co-infections may interact in complex ways, sometimes resulting in competitive suppression between parasites, and other times potentially enhancing overall virulence, highlighting the importance of considering these dynamics in ecological and evolutionary analyses.

Alterations in the leukocyte formula. Alteration of the heterophil/lymphocyte (H/L) ratio is considered an indicator of immune response. However, some authors reported no association between changes in the H/L ratio and infection by *Leucocytozoon* spp. [37], *Haemoproteus killangoi*, and *Haemoproteus zosteropis* or *Plasmodium* spp. [59]. This last author, by contrast, reported an increase in this ratio in New Caledonian Zosterops (*Zosterops* spp.) parasitized by microfilariae [59].

On the other hand, other studies reported increases in the numbers of heterophils and lymphocytes in individuals infected with hemosporidia [141]. A higher white blood cell count (WBC) was observed in parasitized barn owls than in non-parasitized individuals [99].

Although the number of heterophils showed a significant decrease in parasitized blackcaps (*Sylvia atricapilla*), no association was found between this parameter and treatment with primaquine or parasitic burden [68]. By contrast, in the same study, lymphocyte counts did not vary throughout the study but were higher in birds with multiple hemoparasitic infections (*Plasmodium* spp., *Haemoproteus* spp., and *Leucocytozoon* spp.). These findings suggest that specific leukocyte populations, such as lymphocytes, could be influenced by the complexity of the parasitic burden, particularly in cases of co-infection. This highlights the potential value of leukocyte profiling in assessing the immune responses to hemoparasitism, although further research is needed to clarify these dynamics.

Alterations in analytes related to oxidative stress. The digestion of hemoglobin by some hemoparasite species leads to iron release, which can catalyze the formation of reactive oxygen metabolites (ROM), recognized markers of cellular oxidative stress. Conversely, total glutathione (tGSH) functions as an endogenous antioxidant and, together with ROM levels, these biomarkers can be used to assess the oxidant/antioxidant balance and the presence or absence of oxidative stress. Some studies suggest that generating free radicals may be responsible for inducing tissue damage [256]. Based on this premise, some authors found a positive association between ROM levels and parasitemia, but not for tGSH in great tits (n = 299) [44]. Moreover, an interaction between sex and tGSH with hemoparasitemia was observed, with infected males showing higher tGSH levels than uninfected males. By contrast, infected females exhibited lower tGSH levels or no significant difference compared to uninfected females [44].

Corticosterone is a key hormone in the stress response [257]. A study on house sparrows (n = 113) assessed the corticosterone levels in captured birds. Two blood samples were collected: one immediately after capture and another 30 min post-capture. Using nested PCR (nPCR) to determine the infection status with *Haemoproteus* spp., *Plasmodium* spp., and *Leucocytozoon* spp., the authors found no significant correlation between hemoparasitemia and either baseline or stress-induced corticosterone levels [79]. These results suggest that hemosporidia infection does not markedly alter hypothalamic–pituitary–adrenal (HPA) axis function in this bird species.

Also, thiobarbituric acid reactive substances (TBARS) produced during lipid peroxidation and oxidation serve as valuable indicators of oxidative stress response. In this sense, birds infected with *Haemoproteus* spp. exhibited significantly higher TBARS levels, probably due to increased oxidative damage that can harm avian health [97].

In great tits (n = 92), the relationship between hemoparasitism and oxidative stress was investigated by measuring four key markers of oxidative stress: pro-oxidant production, such as mitochondrial superoxide production in red blood cells (RBCs); antioxidant defenses, such as plasma antioxidant capacity; oxidative damage, measured by reactive oxygen metabolites in plasma; and RBC membrane resistance to oxidative damage. Superoxide production in red blood cells (RBCs) was higher in infected individuals than in uninfected ones. Although no direct relationship was observed between ROM and infection status, a notable association was found between clutch size, ROM levels, and infection status. In uninfected individuals, ROM levels increased as clutch size increased, while in infected individuals, ROM levels rose as clutch size decreased. No relationship was found between RBC membrane resistance to oxidative attack or plasma antioxidant capacity and infection status [60]. These results suggest that increased superoxide production in infected individuals may be attributed to higher energy demands. This could arise from the activation of immune functions [258], which can be energetically costly, or from the diversion of energy by the parasite for its development [60].

Alterations in inflammatory proteins: immunoglobulins and haptoglobin. In birds, haptoglobin is classified as an acute-phase protein, with its concentration increasing during inflammatory processes. An elevation in haptoglobin levels was observed in parasitized birds (n = 424) from 22 species, suggesting that hemoparasitism (*Plasmodium* spp., *Haemoproteus* spp., or *Leucocytozoon* spp.) could be associated with inflammatory responses [141].

Interestingly, in blackcaps (n = 64) treated with primaquine, IgY levels were positively correlated with parasitic burden (*Haemoproteus* spp., *Plasmodium* spp., or *Leucocytozoon* spp.), decreasing significantly when treatment was applied. Furthermore, when controlling infection intensity, a significant negative correlation was observed between initial plasma immunoglobulin and haptoglobin levels [68].

Across studies, *Plasmodium* spp. infections emerged as more consistently associated with hematological alterations, particularly reductions in hematocrit levels and PCV, compared to *Haemoproteus* spp. or *Leucocytozoon* spp. These changes are likely linked to the parasite’s life cycle, which involves invasion and destruction of red blood cells. By contrast, other hemoparasites may cause subtler or more transient hematological effects. The interplay between parasite species, co-infection status, and host physiology underscores the need for standardized hematological assessments in wildlife parasitology to facilitate cross-study comparisons and early detection of clinically relevant impacts.

### 4.4. Effects on Bird Activity, Body Temperature, and Behavior

This category may be closely associated with the effects of hemoparasitism on oxidative stress, as increased energy expenditure could reflect alterations in host activity, thermoregulation, and behavior. The authors analyzed it separately, given that it primarily has qualitative characteristics. Given the need for a control group for such specific measurements, it is noteworthy that, with one exception, all of the articles analyzed in this category were experimental studies, in which wild birds were captured, and in some cases, *Plasmodium* spp. was inoculated [62,104,154], or the specimens were already infected with *Plasmodium* spp. or *Leucocytozoon* spp. [56]. Reaction time was measured in both a control group and an experimentally infected group, finding that parasitemia itself did not alter the birds’ reaction [62]. On the other hand, parasitized birds exhibited significantly reduced activity as early as the first few days after experimental infection, which could lead to long-term effects on bird behavior. Even when parasitemia decreased considerably, the birds did not fully recover and continued to exhibit reduced activity. However, a trend toward increased activity was established in chronic infections [62,160].

The aerobic performance of migratory birds has been investigated by analyzing the resting metabolic rate (RMR), which represents the energy expenditure to maintain basic functions like breathing, circulation, and organ function under resting conditions. The RMR of uninfected birds remained indistinguishable from that of birds with low parasitemia. However, birds with high parasitemia (infected with *P. relictum*) exhibited a significantly lower RMR during the migratory period than uninfected birds. By contrast, the endurance times of free-living infected birds did not show significant differences compared to their parasite-free conspecifics [104]. Thus, although high parasitemia alters the RMR of migratory birds, the impact on aerobic performance and endurance capacity appears to be less pronounced during migration. Consequently, despite parasitemia, birds in migratory dispositions could maintain demanding exercise levels for considerably long periods. These results highlight the complexity of the relationship between infection and behavior in birds, suggesting that infection status, whether acute or chronic, can have varying impacts on activity levels. Regarding body temperature, infection was related to temperature in approximately half of the predictive models analyzed by Jenkins et al.: in one of the populations examined, infected birds exhibited lower body temperatures than uninfected birds [56]. Although infection influenced body temperature in some models, at the end of the experiment, infection status was not a strong predictor of body temperature, suggesting further investigation of other factors that may influence temperature variation in infected birds.

### 4.5. Effects on Feather Quality or Growth

Few studies analyzed the effect of hemoparasites on bird plumage, both in terms of feather quality and growth. In Western house martins (n = 444), the length of rectrix feathers was shorter in *Haemoproteus* spp.- or *Plasmodium* spp.-infected birds compared to uninfected ones. Additionally, infected birds showed more growth bands on their feathers than uninfected individuals, and the latter exhibited a higher growth rate of rectrix feathers than infected birds [45]. Two hypotheses have been proposed to explain this association. The first one, supported by studies by Sherman et al. [259] and Martin and Kirk [260], suggests that some parasites, such as *Plasmodium*, obtain essential amino acids from the host plasma and digest host hemoglobin. Thus, the shortening of feathers could result from the essential nutrients directly consumed by hemoparasites. The second one considers that differences in daily feather elongation could be related to activation of the immune response. Since both avian molt and the immune response are nutrient-demanding processes, the feather growth rate in infected house martins was reduced, as resources must be reallocated to develop this immune response. However, in studies conducted on *P. relictum*, *Plasmodium ashfordi* [41], and *Haemoproteus majoris* [85] in captured birds, no relationship was found between hemoparasitism and feather growth or quality, highlighting the need for further studies under different conditions to truly understand the consequences of hemoparasitosis on bird plumage.

### 4.6. Interactions with Ectoparasites

Only two articles investigated the association between ectoparasites and the presence or quantity of hemoparasites [31,45]. Both studies were conducted in the same exact location, with the same host (Western house martin) and similar conditions. Despite the similarity in approaches, the results obtained were remarkably diverse. In the first study [31], the authors found that specimens harboring *Haemoproteus* spp. and *Plasmodium* spp. had significantly higher numbers of ectoparasites (chewing lice and feather mites). On the contrary, in the second article [45], there were no significant differences in the abundance of chewing lice between *Haemoproteus* spp.- and *Plasmodium* spp.-infected and uninfected birds. Unlike the first study, hemoparasite sequencing was not performed in the last study, and the detection of dual infections by hemoparasites was impossible. This fact highlights the importance of accurate hemoparasite detection in better understanding the interactions between ectoparasites and hemoparasites. One of the hypotheses put forward by Marzal et al. [31], in finding a relationship between co-infection by hemoparasites and the number of ectoparasites, is that because grooming in birds is an activity that requires considerable time and energy [261], the higher levels of ectoparasites observed in co-infected birds could be interpreted as a consequence of the weakness induced by infection with these hemoparasites.

## 5. Lesions and Mortality

Eighteen studies included in this review analyzed mortality directly associated with the presence of hemoparasites. In captured free-living birds where individuals were assumed to return to the same nest box, survival rates were inferred based on the absence of recapture on the following performances [31,32,39]. Other investigations were conducted on deceased individuals, primarily during mortality outbreaks in penguins [120,206,222,241,243]. The number of specimens analyzed in each study was generally small, limiting the potential for statistical associations. In these cases, the matrix analyzed often consisted of tissue samples with abundant histological descriptions, mainly of the liver, spleen, and lungs. These five articles examined the presence of *Plasmodium* spp., among other hemoparasites, and four reported hepatic alterations. These findings were observed through histopathological analysis or postmortem examination, such as hepatomegaly [120,241] or pinpoint flat white to off-white spots disseminated over the hepatic capsule [241]. The latter revealed a high number of protozoan schizonts and macrophages within hepatic vessels and parenchyma [120,206,241], as well as lipid accumulation in hepatocytes [243]. Additionally, these five studies reported alterations in the spleen, such as splenomegaly [120,222], multifocal necrotic areas within the splenic parenchyma [241], and increased phagocytic activity [120,206,241,243]. Furthermore, some studies also noted pulmonary lesions, including pulmonary edema [120,222], interstitial hypercellularity [243], and pulmonary congestion [206].

As stated above, the number of studies directly linking hemoparasitic infection to mortality rate was limited, primarily due to small sample sizes and the inherent challenges of field-based data collection. Nevertheless, the consistent pathological findings across these investigations highlight a clear association between *Plasmodium* spp. infection and damage to vital organs. The recurring hepatic, splenic, and pulmonary alterations observed underscore the potential severity of hemoparasitic infections, particularly during mortality outbreaks [120,206,222,241,243]. These findings reinforce the importance of incorporating histopathological analysis into postmortem studies to deepen our understanding of the pathological mechanisms and broader health impacts of hemoparasites in avian species.

## 6. Diagnostic Methods

It is often challenging to obtain enough subjects or representative samples to conduct a meaningful study with wild birds, which impedes progress in advancing knowledge in this field. Furthermore, diagnostic techniques are often limited to optical microscopy due to the limited resources available in rehabilitation centers. Optical microscopy may lack high sensitivity, and its specificity can vary depending on the observer’s experience [262]. The development of new diagnostic techniques to detect hemoparasites in recent decades has enabled, among other things, a better understanding of these parasitic infections [262], which is crucial for establishing greater control over them, particularly in tropical and subtropical regions where favorable ecological conditions require sustained surveillance and control measures. Despite these challenges, the growing field of hemoparasitology has begun to address some of these gaps in knowledge. Advances in molecular techniques, such as next-generation sequencing, have allowed for a more detailed understanding of hemoparasite genomes, enabling researchers to explore new aspects of parasite biology and host-parasite interactions. However, much work remains to completely clarify the immune response to hemoparasitic infections, their transmission dynamics, and the broader ecological factors that influence their prevalence and health impact.

To analyze the detection of different hemoparasites, the type of sample collected for analysis in each article was compiled. It was reasonable to assume that blood would be the most significant sample collected, as venipuncture is a safe and accessible method in avian species of nearly all sizes when performed by a trained veterinarian. It causes minimal harm to the animal [263], although some samples were taken directly from heart puncture [107]. Out of the 231 articles analyzed, 194 collected only blood, 13 collected only organs, and 22 collected both blood and organs. Feathers were collected in two of the articles; in one, blood was also collected [41], and in the other, organs were collected [93]. The organ samples included the intestine, muscle, spleen, liver, lungs, and brain, among others. Although using blood as a matrix for hemoparasite analysis demonstrates the ease of sample collection and the opportunity to detect hemoparasites circulating in peripheral blood, hemoparasitic infections may go undetected. Moreover, prevalence may be underestimated in host individuals with chronic infections not undergoing a relapse phase characterized by elevated blood parasitemia [226,264]. Ideally, hemoparasite detection should include the analysis of additional matrices beyond blood, such as internal organs [200].

**Figure 6:**
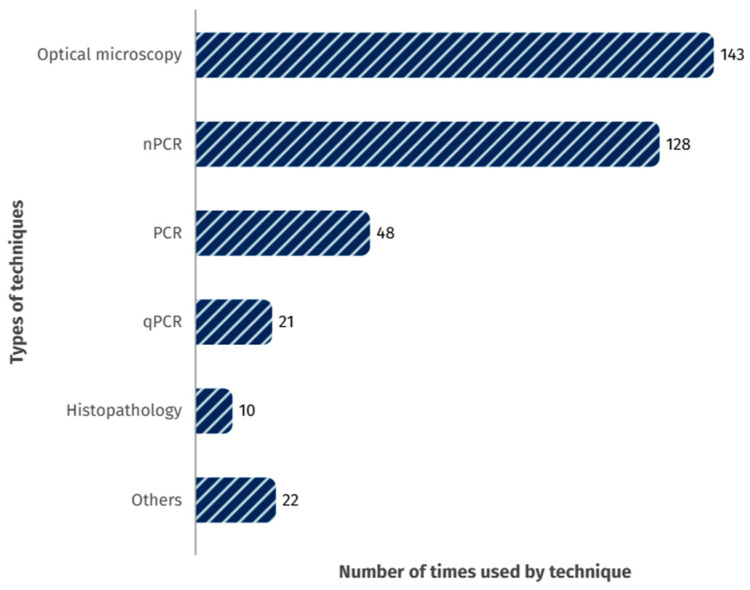
Frequency of use of diagnostic techniques in the reviewed studies. ‘Others’ category includes serology, including ELISA, in situ hybridization (ISH), immunohistochemistry, the buffy coat method, electron microscopy, and genomic sequencing, among others.

The techniques employed with these samples were compiled and divided into the following categories: optical microscopy, nested PCR (nPCR), conventional PCR (PCR), real-time PCR (qPCR), histology or histopathology, enzyme-linked immunosorbent assay (ELISA), chromogenic in situ hybridization (CISH), electron microscopy, and others. The numbers of studies that employed each recorded technique are detailed in Figure 6. Although some studies addressed the variability in hemoparasite detection results across different diagnostic techniques [265,266,267], optical microscopy was the most employed method. The low cost and short processing time of microscopy and the development of various microscopic quantification approaches have contributed to its continued use. Moreover, microscopy has shown comparable effectiveness to molecular techniques such as nPCR in detecting hemoparasites [267].

**Figure 7:**
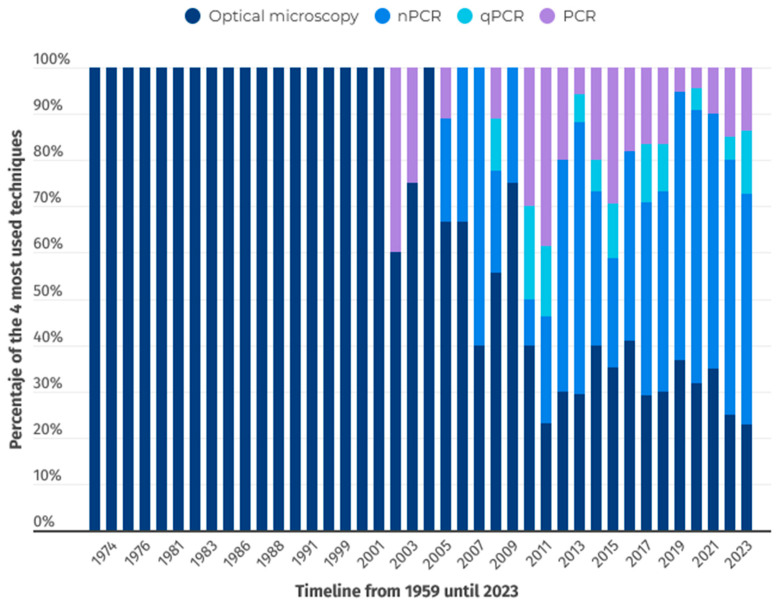
Temporal trends in the use of the four most common diagnostic techniques (1959–2023).

A temporal evolution analysis of the different techniques was designed to better understand the various techniques used for analyzing hemoparasitosis, as shown in Figure 7. Scientific advances in the development of molecular techniques are reflected in their increasing use over time, progressively displacing other diagnostic methods.

**Figure 8:**
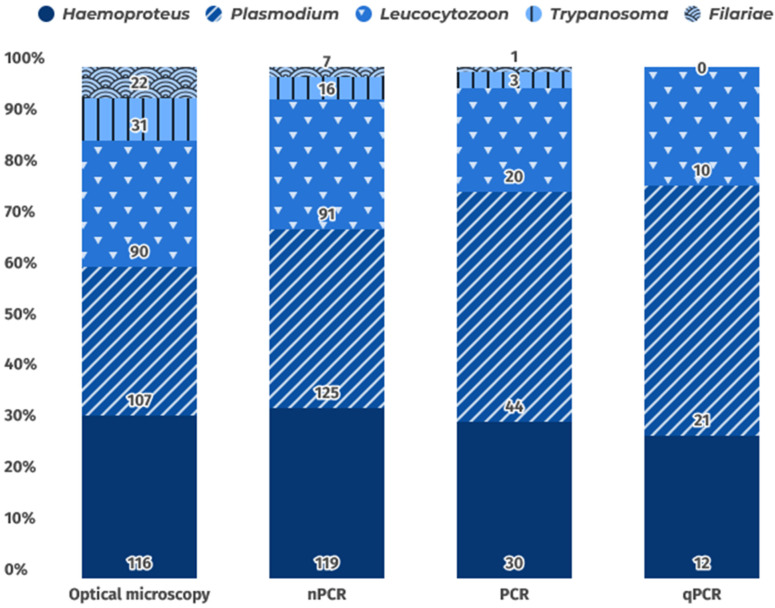
Distribution of the five most frequent hemoparasites detected by the four most employed diagnostic techniques in the reviewed studies.

In this way, to determine the type of technique used for analyzing each hemoparasitosis, data on each of the hemoparasites analyzed with the four most common methods in hemoparasite analysis were gathered: optical microscopy, nPCR, qPCR, and PCR. Figure 8 shows that filarial worm and *Trypanosoma* diagnoses were based mainly on microscopic detection. It should be noted that molecular diagnostic techniques were not employed in the compiled articles until 2002. The nested PCR protocol developed by Hellgren et al. [268] was also widely used, as it allows simultaneous detection of the genera *Leucocytozoon*, *Plasmodium*, and *Haemoproteus*. It is worth noting that the first study to use qPCR to diagnose hemoparasites was published in 2008. Since then, 21 studies have employed this technique, establishing qPCR alongside nested PCR as an increasingly adopted method, gradually replacing conventional PCR for hemoparasite analysis.

Finally, articles in which the parasitic load of hemoparasites was quantified, either by optical microscopy or qPCR, were compiled. Of the 231 articles analyzed, 113 quantified the hemoparasitic load. Notably, there was inequity between the articles that conducted quantification and those that did not. When examining each hemoparasite individually, almost no variation in quantification could be observed between hemoparasites. According to these data, and given that most quantification relied on optical microscopy, this lack of variation could be explained by the fact that quantifying an additional hemoparasite beyond those targeted initially in the study does not represent a significant increase in economic cost for the researcher.

A major limitation identified during this review was the inconsistent reporting of parasite taxonomy and infection metrics across studies, especially in the earlier literature. In many cases, results were presented only at the genus level or aggregated across host species, precluding the generation of a complete matrix of bird species by hemoparasite taxa. Addressing this gap will require standardization of reporting practices to allow future reviews to identify and quantify these associations more precisely.

## 7. Conclusions

This systematic review was focused on hemoparasites and their health impacts on wild birds. Studies remained unevenly distributed across regions, bird groups, and parasite types. Most were descriptive, with few experimental analyses, highlighting the need for such approaches. The health effects of hemoparasites were inconsistent, varying with host species, age, infection intensity, and environment. However, some studies, as is common in wild species research, lacked data about some of these variables, limiting the conclusions of many of them. Nevertheless, authors described a greater pathogenic potential in *Plasmodium* spp. infections compared to *Leucocytozoon* spp. or *Haemoproteus* spp., suggesting species-specific differences in virulence. While some studies linked infections to reduced body condition, immune changes, oxidative stress, or reproduction, many showed no clear impact, reflecting complex host–parasite dynamics. Notably, infection probability appears to increase with host age, likely due to the chronic nature of many hemoparasites, and co-morbidities may influence outcomes, though they were rarely assessed. Moreover, the hemoparasite forms typically detected in blood were gamonts, which appear after the parasite has completed earlier developmental stages in internal organs, where any potential pathogenic effects are more likely to take place, biasing the possible pathogenic effects of hemoparasites. While variability is inherent in wildlife health studies, synthesizing the available evidence revealed certain patterns with practical relevance. For instance, the more pathogenic potential of *Plasmodium* spp. compared to *Leucocytozoon* spp. or *Haemoproteus* spp. was supported across different geographic contexts, particularly when infection intensity was high or when co-infections occurred. These patterns highlight the importance of integrating hemoparasite monitoring into conservation and disease surveillance programs, especially in regions undergoing rapid environmental change where shifts in vector distributions may alter host–parasite dynamics. Finally, diagnostic challenges were detected, especially reliance on microscopy in low-resource settings and limited detection. To advance understanding, there is a need for better diagnostic techniques, standardized methods, and expanded research in understudied areas and taxa. Future work should focus on longitudinal and interdisciplinary studies to clarify hemoparasitic effects on wild bird populations.

## Data Availability

The data supporting this systematic review are available upon request from the corresponding author.

## Supplementary Materials

The following supporting information can be downloaded at: https://www.mdpi.com/article/10.3390/ani15172570/s1, Supplementary Table S1: Number of published articles assessing the presence of haemosporidian parasites in different bird species, classified by order and family. Supplementary Table S2: Overview of reported effects of avian hemoparasitism across previous studies, including impacts on the three most studied categories. References [31,37,44,45,52,56,59,60,62,68,72,73,75,76,77,79,85,89,90,97,99,105,111,135,136,141,146,152,159,160,161,176,181,214,240,244,247] are cited in the Supplementary Materials. References numbers cited in the Supplementary Table S2 correspond to the the Main Text Reference Section.
