# Supplementary material for: Hemoparasites in Wild Birds: A Systematic Review of Their Ecology and Clinical Implications
Source: Animals (Basel). 2025 Sep 1;15(17):2570. doi: 10.3390/ani15172570 (PMC12427399; doi:10.3390/ani15172570)
Supplement: Supplementary file 1 [file animals-15-02570-s001.zip › animals-3810566-supplementary/animals-3810566-supplementary Table S2.pdf]

Reference	Reference number*	N	Host	Hemoparasite	Effects on body condition, mass, and growth
Forrester *et al*., 1980	[111]	19	Wild turkey (*Meleagris gallopavo*)	*Plasmodium hermani*	Growth was slower in infected wild poults compared to their uninfected controls.
Marzal *et al*., 2008	[31]	3818	House martins (*Delichon urbicum*)	*Haemoproteus* spp. and *Plasmodium* spp.	Double-infected birds had lower body mass than single-infected or uninfected individuals.
Karell *et al*., 2011	[37]	111	Tawny owl (Strix aluco)	*Leucocytozoon* spp.	*Leucocytozoon* infection did not directly affect body mass. Still, an interaction between colour morph and *Leucocytozoon* infection revealed that *Leucocytozoon* infection was associated with lower body mass in the brown morph but not in the grey morph.
Baillie *et al*., 2012	[240]	384	New Zealand bellbird (*Anthornis melanura*)	*Plasmodium* spp.	Significant negative relationship between body condition and infection at one location (Hauturu) and a significant positive relationship at another (Tawharanui).
Grillo *et al*., 2012	[135]	187	Prothonotary warbler (*Protonotaria citrea*)	*Haemoproteus* spp. and *Plasmodium* spp.	A robust correlation exists between nestling age and mean nestling mass, regardless of the infection status of the female.
Isaksson *et al*., 2013	[44]	299	Great tit (*Parus major*)	*Plasmodium relictum* and *Plasmodium ciricumflexum*	There were no significant associations between species-specific parasitaemia and any of the physiological variables studied.
Marzal *et al*., 2013	[45]	444	House martins (Delichon urbicum)	*Haemoproteus* spp. and *Plasmodium* spp.	Infected house martins had lower body mass than uninfected individuals.
Jenkins *et al*., 2015	[56]	65	Great tit (*Parus major*)	*Leucocytozoon* spp. and *Plasmodium* spp.	When birds suffered the highest infection intensity, there was no consistent effect of infection on body condition.

Meixell *et al*., 2016	[247]	1347	Eurasian teal (*Anas crecca*), American wigeon (*Anas americana*), mallard (*Anas platyrhynchos*), Northern pintail (*Anas acuta*), Lesser Scaup (*Athya affinis*).	*Haemoproteus* spp., *Plasmodium* spp. and *Leucocytozoon* spp.	The BCI effect for *Haemoproteus* was negative for juvenile birds and weakly negative for adults. For L*eucocytozoon*, the BCI effect varied by species, going through negative, positive, or equivocal depending on each species. No support was found for variation in prevalence relative to interactions with co-infection status and BCI, and prevalence related to Plasmodium infection.
Mukhin *et al*., 2016	[62]	20	Eurasian siskin (*Spinus spinus*)	*Haemoproteus* spp., *Plasmodium* spp.* and *Leucocytozoon* spp.	Experimental infection (*Plasmodium relictum*) did not lead to weight loss.
Sijbranda *et al*., 2017	[244]	201	14 species	*Plasmodium* spp.	No significant relationship was found between total parasite load and BCI in blackbirds, silvereyes, or NZ robins.
Arriero *et al*., 2018	[68]	64	Eurasian blackcap (*Sylvia atricapilla*)	*Haemoproteus* spp., *Plasmodium* spp. and *Leucocytozoon* spp.	The medication treatment and body condition were not associated with IgY levels, and we did not see differences between sexes or between individuals with single or multiple infections, irrespective of the treatment.
Hahn *et al*., 2018	[105]	124	Great Reed Warbler (Acrocephalus arundinaceus)	*Haemoproteus* spp. and *Plasmodium* spp.	Parasitaemia did not affect any of these morphological variables at any stage we sampled: fat stores and pectoral muscle size.
Pigeault *et al*., 2018	[72]	1181	Great tit (*Parus major*)	*Haemoproteus* spp., *Plasmodium* spp. and *Leucocytozoon* spp.	No effect of infection status was observed on BCI.
Townsend *et al*., 2018	[152]	240	American crow (*Corvus brachyrhynchos*)	*Haemoproteus* spp., *Plasmodium* spp. and *Leucocytozoon* spp.	BCI did not vary with the prevalence or burden of any parasite.

Cadena-Ortiz *et al.*, 2019	[214]	871	36 species	*Haemoproteus* spp. and *Plasmodium* spp.	No relationship between parasite lineage and BCI was observed.
Ilgūnas *et al.*, 2019a	[75]	32	Reed warbler (Acrocephalus scirpaceus), Common starling (Sturnus vulgaris), Red crossbill (Loxia curvirostra)	*Haemoproteus* spp., *Plasmodium* spp.* and *Leucocytozoon* spp.	No significant changes between the body mass of the experimental and control groups of Common crossbills were observed during the experiment.
Jiménez-Peñuela *et al.*, 2019	[77]	45	House sparrow (*Passer domesticus*)	*Haemoproteus* spp., *Plasmodium* spp. and *Leucocytozoon* spp.	Bird body condition index (BCI) decreased with increasing urban land cover in both yearling and adult birds from urban habitats. In urban yearlings, BCI increased with *Haemoproteus* or *Plasmodium* infection, whereas in urban adults, BCI was unrelated to haemosporidian infection. In rural and natural habitats, the BCI of both adults and yearlings was not associated with land cover or infection status. Additionally, *Plasmodium*-infected urban birds showed lower body mass variance compared to uninfected individuals.
Bichet *et al.*, 2020	[79]	113	House sparrow (*Passer domesticus*)	*Haemoproteus* spp., *Plasmodium* spp. and *Leucocytozoon* spp.	Infection status was not associated with sparrow condition in general, but infected juveniles had a higher body condition than uninfected juveniles.
Gupta *et al.*, 2020	[181]	1177	28 species	*Haemoproteus* spp., *Plasmodium* spp. and *Leucocytozoon* spp.	*Haemoproteus* prevalence increased significantly with birds having better BCI.
Huang *et al.*, 2020	[85]	1803	62 species	*Haemoproteus* spp., *Plasmodium* spp. and *Leucocytozoon* spp.	For hatching year individuals, there were no significant relationships between infection status and body mass.

Aželytė *et al.*, 2022	[90]	30	Common rosefinch (*Carpodacus erythrinus*), Sedge warbler (*Acrocephalus schoenobaenus*), Eurasian siskin (*Spinus spinus*)	*Plasmodium relictum*	In neither of the groups was there any significant correlation determined between the parasitemia and body mass variation
Pacheco *et al.*, 2022	[159]	87	Great-tailed grackle (*Quiscalus mexicanus*)	*Plasmodium* spp., and *Haemoproteus* spp.	*Plasmodium* infection was not associated with differences in body weight and BCI for males and females.
Jiménez-Peñuela *et al.*, 2023	[97]	688	House sparrow (*Passer domesticus*)	*Haemoproteus* spp., *Plasmodium* spp. and *Leucocytozoon* spp.	The relationship between TBARS and body condition was negative for *Haemoproteus-infected birds* and negative for uninfected birds.
Martín-Maldonado *et al.*, 2023	[99]	134	Barn owl (*Tyto alba*), Eurasian eagle-owl (*Bubo bubo*), Tawny owl (*Strix aluco*), Little owl (*Athene noctua*), Eurasian scops owl (*Otus scops*), Long-eared owl (*Asio otus*)	*Haemoproteus* spp., *Plasmodium* spp., *Leucocytozoon* spp., *Trypanosoma* spp. and microfilarial worms	The presence of hemoparasites was more frequent in individuals with a lower BCI. The presence of hemoparasites is five times more likely in barn owls with a low BCI than in barn owls with a standard BCI.
Talbott *et al.*, 2023	[160]	26	Dark-eyed junco (*Junco hyemalis hyemalis*)	*Haemoproteus* spp. and *Plasmodium* spp.	We detected a trend toward higher BCI in birds with chronic infections. A model with the same predictors for BCI showed an increase in BCI from baseline to post-inoculation in experimentally inoculated birds. In contrast, baseline infection status did not affect BCI differentials.

* References numbers correspond to the the Main Text Reference Section.

Reference	Reference number*	N	Host	Hemoparasite	Effects on reproductive success and survival
Marzal *et al*., 2008	[31]	3818	House martins (*Delichon urbicum*)	*Haemoproteus* spp. and *Plasmodium* spp.	There were no differences in survival probability between double and single-infected house martins. Clutch size of individuals with double infection was significantly larger than that of single-infected and uninfected house martins. Double-infected individuals produced a significantly higher number of fledglings than single-infected ones.
Grillo *et al*., 2012	[135]	187	Prothonotary warbler (*Protonotaria citrea*)	*Haemoproteus* spp. and *Plasmodium* spp.	Upon comparing infected and uninfected females, there were no significant differences in any of the reproductive parameters. No significant differences were found in the reproductive fitness of uninfected and infected older and younger females.
Marzal *et al*., 2013	[45]	444	House martins (Delichon urbicum)	*Haemoproteus* spp. and *Plasmodium* spp.	Uninfected birds laid larger clutches and initiated earlier than infected birds. They also had higher breeding success and fledged more nestlings than infected birds.
Asghar *et al*., 2015	[52]	75	Great reed warbler (*Acrocephalus arundinaceus*)	*Haemoproteus* spp. and *Plasmodium* spp.	Malaria infection reduces lifespan and offspring number; hemoparasitism increases telomere shortening.

Zylberberg *et al*., 2015	[146]	735	White-crowned sparrow (*Zonotrichia leucophrys*)	*Haemoproteus beckeri* and *Plasmodium polare*	Females infected with *H. beckeri* exhibited increases in lifetime reproductive success compared with uninfected individuals. Also, *H. beckeri*-infected females laid and hatched about twice as many eggs and fledged about twice as many chicks as uninfected females. By contrast, *H. beckeri* infection was not associated with lifetime reproductive effort or success of males, nor was *P. polare* infection associated with lifetime reproductive effort or success in either females or males.
Delhaye *et al*., 2016	[60]	92	Great tit (*Parus major*)	*Plasmodium* spp.	Neither *Plasmodium* infection status nor its interaction with reproductive effort, age, or sex affected RBC membrane resistance.
Pigeault *et al*., 2018	[72]	1181	Great tit (*Parus major*)	*Haemoproteus* spp., *Plasmodium* spp. and *Leucocytozoon* spp.	There was no effect of haemosporidian infections on clutch sizes or the number of chicks hatched. Only hosts single-infected by *Haemoproteus* or *Leucocytozoon* had fewer chicks fledged than co-infected birds. No difference between birds single-infected by *Plasmodium* and birds co-infected was observed. Co-infected birds had a lower survival rate than single-infected ones.
Townsend *et al*., 2018	[152]	240	American crow (*Corvus brachyrhynchos*)	*Haemoproteus* spp., *Plasmodium* spp. and *Leucocytozoon* spp.	*Plasmodium* prevalence was associated with lower fledging success. No other direct links with infection were apparent. Fledging success was not linked to *Haemoproteus* or *Leucocytozoon* infections. After fledging, apparent survival was lower for birds infected with Plasmodium as nestlings, but did not vary with other hemosporidian infections.

Dadam *et al*., 2019	[73]	271	House sparrow (*Passer domesticus*)	*Haemoproteus* spp., *Plasmodium* spp., *Leucocytozoon* spp. and *Atoxoplasma* spp.	Sparrow survival was negatively related to the intensity of *Plasmodium* infection for both adult and juvenile sparrows, although the intensity of infection in adults was lower than in juveniles. For *Atoxoplasma* infection intensity, the relationship between infection intensity and survival was insignificant.
Ilgūnas *et al*., 2019b	[76]	64	House sparrow (Passer domesticus), Chaffinch (Fringilla coelebs), Red crossbill (Loxia curvirostra), Common starling (Sturnus vulgaris)	*Haemoproteus* spp., *Plasmodium* spp.* and *Leucocytozoon* spp.	There was no significant difference in mortality in the control and experimental groups.
Pacheco *et al*., 2022	[159]	87	Great-tailed grackle (*Quiscalus mexicanus*)	*Haemoproteus* spp., *Plasmodium* spp. and *Leucocytozoon* spp.	There was no association between being infected and the host sex
Talbott *et al*., 2023	[160]	26	Dark-eyed junco (*Junco hyemalis hyemalis*)	*Haemoproteus* spp. and *Plasmodium* spp.	Sperm count at three weeks post-inoculation and deformed sperm proportion at three weeks post-inoculation were not significant.

* References numbers correspond to the the Main Text Reference Section.

Reference	Reference number*	N	Host	Hemoparasite	Blood parameters and immune response alterations and immune response alterations
Forrester *et al*., 1980	[111]	19	Wild turkey (*Meleagris gallopavo*)	*Plasmodium hermani*	The mean PCV dropped to 20% on day 25 post-infection, and the peak of parasitemia occurred on day 18 for poults infected at 12 to 18 h of age. The results were similar in poults infected at 3 to 5 days of age.
Marzal *et al*., 2008	[31]	3818	House martins (*Delichon urbicum*)	*Haemoproteus* spp. and *Plasmodium* spp.	Double-infected house martins showed the highest haematocrit levels.
Karell *et al*., 2011	[37]	111	Tawny owl (Strix aluco)	*Leucocytozoon* spp.	*Leucocytozoon* infection did not have an overall effect on the Heterophil-lymphocyte ratio.
Astudillo *et al*., 2013	[136]	786	Carolina wren (*Thryothorus ludovicianus*), Indigo bunting (*Passerina cyanea*), Northern cardinal (*Cardinalis cardinalis*), Tufted titmouse (*Baeolophus bicolor*), White-throated sparrow (*Zonotrichia albicollis*)	*Haemoproteus* spp., *Plasmodium* spp., *Leucocytozoon* spp., *Trypanosoma* spp. and microfilarial worms	Average PCV and polychromasia levels were similar between infected and non-infected birds.
Isaksson *et al*., 2013	[44]	299	Great tit (*Parus major*)	*Plasmodium relictum* and *Plasmodium ciricumflexum*	Positive association between ROM and parasitaemia, but not for tGSH.
Ellis *et al*., 2014	[141]	424	22 species	*Haemoproteus* spp., *Plasmodium* spp. and *Leucocytozoon* spp.	Heterophils, lymphocytes, and haptoglobin were elevated in haemosporidian-infected individuals. White blood cell levels did not differ significantly between individuals infected with one or the other lineage.

Jenkins *et al*., 2015	[56]	65	Great tit (*Parus major*)	*Leucocytozoon* spp. and *Plasmodium* spp.	When birds suffered the highest infection intensity, there was no consistent effect of infection on haematocrit or oxidative stress level; infected birds in one (Dorigny) of the two locations studied had lower haematocrit than uninfected ones.
Clark *et al*., 2016	[59]	449	Zosterops (*Zosterops* spp.)	Haemoproteus spp., Plasmodium spp. and Microfilarial worms	Microfilariae were associated with increased H/L ratios when accounting for time and presence of other parasites. Neither *Haemoproteus* nor *Plasmodium* spp. Influenced H/L ratios, either as separate variables or combined
Delhaye *et al*., 2016	[60]	92	Great tit (*Parus major*)	*Plasmodium* spp.	RBC superoxide production was higher in infected individuals compared to uninfected ones. There was no effect of infection per se on ROM quantity. Plasma antioxidant capacity was not correlated with parasitaemia. Neither *Plasmodium* infection status nor its interaction with reproductive effort, age, or sex affected RBC membrane resistance.
Sijbranda *et al*., 2017	[244]	201	14 species	*Plasmodium* spp.	Robins infected with *Plasmodium* spp. showed a significantly lower PCV than robins that were uninfected. No significant relationship was found between total parasite load and PCV in blackbirds, silvereyes, or NZ robins.
Arriero *et al*., 2018	[68]	64	Eurasian blackcap (*Sylvia atricapilla*)	*Haemoproteus* spp., *Plasmodium* spp. and *Leucocytozoon* spp.	IgY levels were positively associated with the intensity of infection and dropped significantly throughout the study. We observed a significant negative correlation between plasma immunoglobulin and haptoglobin in the initial sample while controlling for intensity of infection. Parasite burden was not associated with the medication treatment or intensity of infection, but showed a significant decrease throughout the study. Lymphocyte numbers did not vary significantly

					throughout the study, but were higher in birds with multiple infections.
Hahn *et al*., 2018	[105]	124	great reed warbler (Acrocephalus arundinaceus)	*Haemoproteus* spp. and *Plasmodium* spp.	Haemoglobin concentration did not differ between infected and non-infected captive birds.
Ishtiaq & Barve, 2018	[176]	573	18 species	*Haemoproteus* spp., *Plasmodium* spp. and *Leucocytozoon* spp.	The probability of infection with *Plasmodium* spp. showed significant increases with PCV. However, neither *Haemoproteus* spp. nor *Leucocytozoon* spp. Showed significant variation in infection status with PCV
Townsend *et al*., 2018	[152]	240	American crow (*Corvus brachyrhynchos*)	*Haemoproteus* spp., *Plasmodium* spp. and *Leucocytozoon* spp.	We observed no statistical correlation or statistically significant results with hemoparasites and biochemistry and haematological values.
Ilgūnas *et al*., 2019b	[76]	64	House sparrow (Passer domesticus), Chaffinch (Fringilla coelebs), Red crossbill (Loxia curvirostra), Common starling (Sturnus vulgaris)	*Haemoproteus* spp., *Plasmodium* spp.* and *Leucocytozoon* spp.	Experimental infection had a significant negative effect on the average haematocrit value of common crossbills (p<0.05)
Bichet *et al*., 2020	[79]	113	House sparrow (*Passer domesticus*)	*Haemoproteus* spp., *Plasmodium* spp. and *Leucocytozoon* spp.	Infection status was not associated with the three physiological parameters investigated (haematocrit, baseline corticosterone levels, and stress-induced corticosterone levels).

Wiegmann *et al*., 2021	[89]	528	Common buzzard (*Buteo buteo*), Red kite (*Milvus milvus*), Northern goshawk (*Accipiter gentilis*)	*Haemoproteus* spp., *Plasmodium* spp., *Leucocytozoon* spp., *Trypanosoma* spp. and microfilarial worms	Infection was significantly associated with elevated heterophil, AST, LDH, BA, and BuChE, but decreased lymphocyte and monocyte values.
Aželytė *et al*., 2022	[90]	30	Common rosefinch (*Carpodacus erythrinus*), Sedge warbler (*Acrocephalus schoenobaenus*), Eurasian siskin (*Spinus spinus*)	*Plasmodium relictum*	No significant changes in the hematocrit values occurred throughout the experiment in any of the groups.
Jiménez-Peñuela *et al*., 2023	[97]	688	House sparrow (*Passer domesticus*)	*Haemoproteus* spp., *Plasmodium* spp. and *Leucocytozoon* spp.	We found that birds infected by *Haemoproteus* showed significantly higher TBARS levels than uninfected birds.
Martín-Maldonado *et al*., 2023	[99]	134	Barn owl (*Tyto alba*), Eurasian eagle-owl (*Bubo bubo*), Tawny owl (*Strix aluco*), Little owl (*Athene noctua*), Eurasian scops owl (*Otus scops*), Long-eared owl (*Asio otus*)	*Haemoproteus* spp., *Plasmodium* spp., *Leucocytozoon* spp., *Trypanosoma* spp. and microfilarial worms	WBC count was significantly higher in parasitized barn owls than in non-parasitized birds. In the Eurasian eagle-owl, no association among the parasitemia of each parasite genus and the hematological parameters was detected.
Talbott *et al*., 2023	[160]	26	Dark-eyed junco (*Junco hyemalis hyemalis*)	*Haemoproteus* spp. and *Plasmodium* spp.	Before experimental inoculation, hematocrit values were higher in chronically infected birds than in those without chronic infections. *Plasmodium* experimentally inoculated birds experienced a decline in hematocrit, while baseline infection status did not influence hematocrit differentials.

Kelly *et al*., 2024	[161]	24	House sparrow (*Passer domesticus*)	*Haemoproteus* spp. and *Plasmodium* spp.	Any HPA axis nor immune measures predict parasite load. No HPA axis trait was correlated with any of the immune measures we assessed. Infected sparrows significantly increased the strength of corticosterone negative feedback after malaria infection, but shams and resistant sparrows did not. Infected sparrows but not shams increased the concentration of circulating IgY after malaria infection. When examining post-inoculation groups, HPA axis function, circulating glucose, and most immune measures did not differ between sham, resistant, and infected sparrows, except that infected sparrows had higher expression of the anti-inflammatory cytokine IL-10 in the liver than shams. The only physiological trait predicting parasite load of infected sparrows that out-ranked the null model was the expression of the proinflammatory cytokine TNF-α in the liver. A linear model revealed a significant positive relationship between parasite load and TNF-α expression in the liver. The proportion of lymphocytes increased after inoculation in infected sparrows, but not in sham birds.

* References numbers correspond to the the Main Text Reference Section.
